# Insights into taxonomy and phylogenetic relationships of eleven *Aristolochia* species based on chloroplast genome

**DOI:** 10.3389/fpls.2023.1119041

**Published:** 2023-02-13

**Authors:** Xuanjiao Bai, Gang Wang, Ying Ren, Yuying Su, Jinping Han

**Affiliations:** Institute of Medicinal Plant Development, Chinese Academy of Medical Sciences & Peking Union Medical College, Beijing, China

**Keywords:** Aristolochia, taxonomy, phylogenetic relationship, comparative analysis, chloroplast genome

## Abstract

**Introduction:**

The Aristolochia, as an important genus comprised of over 400 species, has attracted much interest because of its unique chemical and pharmacological properties. However, the intrageneric taxonomy and species identification within *Aristolochia* have long been difficult because of the complexity of their morphological variations and lack of high-resolution molecular markers.

**Methods:**

In this study, we sampled 11 species of *Aristolochia* collected from distinct habitats in China, and sequenced their complete chloroplast (cp) genomes.

**Results:**

The 11 cp genomes of *Aristolochia* ranged in size from 159,375bp (*A. tagala*) to 160,626 bp (*A. tubiflora*), each containing a large single-copy (LSC) region (88,914-90,251 bp), a small single-copy (SSC) region (19,311-19,917 bp), and a pair of inverted repeats (IR) (25,175-25,698 bp). These cp genomes contained 130-131 genes each, including 85 protein-coding genes (CDS), 8 ribosomal RNA genes, and 37-38 transfer RNA genes. In addition, the four types of repeats (forward, palindromic, reverse, and complement repeats) were examined in *Aristolochia* species. *A. littoralis* had the highest number of repeats (168), while *A. tagala* had the lowest number (42). The total number of simple sequence repeats (SSRs) is at least 99 in *A. kwangsiensis*, and, at most, 161 in *A. gigantea*. Interestingly, we detected eleven highly mutational hotspot regions, including six gene regions (*clpP, matK, ndhF, psbT, rps16, trnK-*UUU) and five intergenic spacer regions (*ccsA-ndhD, psbZ-trnG*-GCC*, rpl33-rps18, rps16-trnQ*-UUG*, trnS*-GCU*-trnG*-UCC). The phylogenetic analysis based on the 72 protein-coding genes showed that 11 *Aristolochia* species were divided into two clades which strongly supported the generic segregates of the subgenus *Aristolochia* and *Siphisia*.

**Discussion:**

This research will provide the basis for the classification, identification, and phylogeny of medicinal plants of Aristolochiaceae.

## 1 Introduction

*Aristolochia*, a type genus of the family Aristolochiaceae, is widely distributed in tropical, subtropical, and temperate areas. Approximately 45 species are distributed in China, and 33 are endemic (Huang et al., 2003). Many species of *Aristolochia* possess a long history of medicinal value. For example, *A. manshuriensis* was commonly used as a traditional Chinese medicine to alleviate pathogenic fire. The dry mature fruits of *A. contorta* and *A. debilis* were called “Fructus Aristolochiae” and had been used to relieve cough and alleviate hemorrhoids. Else species such as *A. fangchi*, *A. tagala*, and *A. kwangsiensis* are widely used in folk medicine and are important medicinal plants. However, the outbreak of renal disease among the group of young women who followed the same slimming medicine containing *A. fangchi* sounds an alarm about the delayed toxic effects of *Aristolochia* species (Vanherweghem et al., 1993; Tomlinson et al., 2020). After decades of investigation, increasing research verified the aristolochic acid contained in the *Aristolochia* species was the main causative factor of nephropathy and may be the potential to cause cancer (Stefanovic et al., 2006; Jelaković et al., 2019). Hence, the *Aristolochia* species have been excluded from the Chinese pharmacopeia and banned to utilize for medicinal purposes in many countries (Kim and Lim, 2019). Yet the conflict between the medicinal value and potential nephrotoxicity and teratogenicity makes the illegal addition of *Aristolochia* in medicines and health products still rampant (Maggini et al., 2018; Ji et al., 2021). Recently, modern studies gradually discovered the new bioactivities of *Aristolochia* species such as insecticidal, anti-bacterial, anti-nociceptive, and anti-inflammatory effects (Kuo et al., 2011; Salome et al., 2020). Therefore, the strict supervision and accurate utilization of the *Aristolochia* species are important to implement the medicinal value.

Elucidating the relationships between species of genus *Aristolochia* is crucial for understanding and harnessing the medicinal properties of the different species. However, as a diverse genus with a large number of species distributed widely in geography, the circumscription and infrageneric classification of genus *Aristolochia* have been complicated and ambiguous. In the cladistic analysis based on morphological characters, many infrageneric taxa have been recognized by different authors (Ohi-Toma and Murata, 2016). For example, González et al. proposed that genus *Aristolochia* should be divided into three subgenera (*Aristolochia*, *Pararistolochia* and *Siphisia*), while Stevenson et al. indicated that the genus consisted of four genera in two subtribes Aristolochiinae and Isotrematinae (González and Rudall, 2003; Buchwalder et al., 2014; Ohi-Toma and Murata, 2016). Besides, in the *Flora of China*, it is also stated a controversy that some species of *Aristolochia* should be transferred to the genus *Isotrema* (Huang et al., 2003). Molecular markers are a reliable alternative that is independent of morphological feathers, enabling them to address the taxonomic challenges arising from the blurring morphological characters (Wu et al., 2020). Numerous molecular methods have been applied to *Aristolochia* and have advanced the understanding of the relationships of the genus *Aristolochia* (Wanke et al., 2006; González et al., 2014; Zhao et al., 2021). The phylogenetic trees produced with three gene sequences *rbcL*, *phyA* and *matk* of *Aristolochia* supported that *Aristolochia* was composed of two lineages corresponding to Aristolochiinae and Isotrematinae, respectively (Ohi-Toma et al., 2006). Based on the combined analysis using two plastid genic spacers (*rps16-trnK* and *petB-petD*) and two nuclear genes (*phyA* and ITS2), the phylogeny construction results confirmed that genus *Aristolochia* was divided into two well-supported clades representing subtribe Aristolochiinae and Isotrematinae, and Zhu et al. suggested *Aristolochia* subgenus *Siphisia* should be treated as an independent genus *Isotrema* (Zhu et al., 2019a). However, the results of different studies are not completely consistent, and the taxonomic systems of *Aristolochia* are still controversial (Wanke et al., 2006; Buchwalder et al., 2014; Ohi-Toma and Murata, 2016). With the new values and species of *Aristolochia* gradually published, effective methods to resolve the phylogenetic relationships and assess the previous classification of *Aristolochia* species are urgently needed (Yang et al., 2018; Luo et al., 2020).

With the rapid development of next-generation DNA sequencing (NGS) technologies, obtaining a complete plastome sequence has become a laboratory routine (Shi et al., 2019). The complete chloroplast (cp) genomes, as the important organelle DNA in plants, are characterized by a large size, containing richer variant site information to be an attractive tool for phylogenetic studies of plants (Niu et al., 2018). Compared with the phylogenetic analysis based on the limited phylogenetic information provided by short fragments of nuclear and cp DNA, the cp genome has significant advantages in phylogenetic resolution, particularly at low taxonomic levels (Parks et al., 2009; Wilkinson et al., 2017; Wu et al., 2021). For example, plastid genome data provided strong support for the sister relationship of sect. *Macroceras* and sect. *Diphyllon* of the genus *Epimedium* (Guo et al., 2022). In recent years, several cp genomes of *Aristolochia* have been reported (Kim and Lim, 2019; Zhao et al., 2021). The molecular structure and phylogenetic analyses of cp genomes of *Aristolochia debilis* and *Aristolochia contorta* revealed a close phylogenetic relationship with Piperaceae, Laurales, and Magnoliales (Zhou et al., 2017). Nevertheless, the compared analysis of multiple *Aristolochia* chloroplasts is still deficient, which is unable to comprehensively illustrate the intricate phylogenetic relationships and systematic evolution of *Aristolochia*.

In this study, we reported eleven complete *Aristolochia* cp genomes including five of subgenera *Siphisia* (*A. fulvicoma, A. hainanensis, A. griffithii, A. kwangsiensis* and *A. dabieshanensis*), three in subgenera *Aristolochia* (*A. tagala*, *A. debilis, A. tubiflora*) and another three species (*A. gigantea*, *A. littoralis, A. neolongifolia*) with unclear subgenera information. The comparative genomic analyses were conducted to explore the features and structural differentiation of the sequences. Analysis of simple sequence repeats (SSRs) could screen out potential molecular polymorphic markers for analyzing the genetic diversity and structure of *Aristolochia* populations in the future. Highly variable regions would provide candidate DNA barcodes for further studying *Aristolochia* species identification. Phylogenetic analysis performed by constructing phylogenetic trees enabled to reveal the interspecific relationship of *Aristolochia* species. This study enriched the valuable complete cp genome resources of *Aristolochia* and will contribute to further research on the identification and phylogenetic relationships within the species of the genus *Aristolochia*.

## 2 Materials and methods

### 2.1 Taxon sampling and DNA extraction

Eleven species of *Aristolochia* were newly collected from the Hainan, Yunnan, Xizang, Guizhou, and Hubei Provinces of China (**Supplementary Table 1**). Thereinto, referring to the *Flora of China* (http://www.iplant.cn/), five species (*A. fulvicoma, A. hainanensis, A. griffithii, A. kwangsiensis* and *A. dabieshanensis*) were divided into subgenus *Siphisia*, and other three species of *A. tagala*, *A. debilis* and *A. tubiflora* were recorded in subgenus *Aristolochia*. Besides, three species without taxonomic information on subgenus, *A. gigantea*, *A. littoralis*, and *A. neolongifolia*, were collected to explore the phylogeny. The 11 individuals were frozen at -80°C and the total genomic DNA was isolated from fresh leaves using the Plant Genomic DNA Kit (TIANGEN, Beijing, China) by the manufacturer’s instructions. DNA integrity was examined by electrophoresis in 1% (w/v) agarose gel and their concentration was measured using a NanoDrop 2000C spectrophotometer (Thermo Scientific; Waltham, MA, USA).

### 2.2 DNA sequencing, assembly and annotation

The quantified DNA was used to construct shotgun libraries with insert sizes of 300~500bp and a paired-end library was constructed by TruSeq™ Nano DNA Sample Prep Kit (Illumina, San Diego, CA, USA). Then paired-end sequencing was performed to obtain 150 bp sequences at both ends of each read according to the manufacturer’s manual for the Illumina NovaSeq platform (Illumina, San Diego, CA, USA). Low-quality regions in the original data were trimmed using the software Trimmomatic (Bolger et al., 2014). Then the clean cp reads were screened and compared with the *Aristolochia* sequences published at the National Centre for Biological Information. SOAPdenovo 2 was used to splice the extracted reads into several contigs (Luo et al., 2012). The assembled contigs were connected to cp genome sequences by using the NOVOPlasty (Dierckxsens et al., 2017), and gaps were filled by the GapCloser module in SOAP package. Lastly, the genes, introns and boundaries of coding regions were compared with reference sequences, *A. debilis* (NC036153), and assembled into complete cp genomes. Genome annotation was performed referring to the complete cp genomes of *Aristolochia* and corrected manually. All of the annotated genomes were deposited in GenBank with the accession numbers listed in **Supplementary Table 1**.

### 2.3 Genome structure analyses

Chloroplast circular maps were drawn in OGDRAW v1.3.1 (http://ogdraw.mpimp-golm.mpg.de/) according to the adjusted genome annotation. The GC content was analyzed using MEGA (Tamura et al., 2013). The SSRs were identified by MISA software (Beier et al., 2017) with the thresholds of 10, 5, 4, 3, 3, and 3 repeat units for mono-, di-, tri-, tetra-, penta-, and hexanucleotides, respectively. To identify the long repeat motifs, REPuter (Kurtz et al., 2001) was used to locate direct, reverse, complementary and palindromic sequences, with a minimum repeat size of 30bp and Hamming distance of 3. Statistical analysis was accomplished by GraphPad Prism (GraphPad Sofware, La Jolla, CA, USA).

### 2.4 Comparative and phylogenetic analyses

The whole-genome alignment for the 11 *Aristolochia* cp genomes was performed and plotted using mVISTA software (Dubchak and Ryaboy, 2006). Comparison of boundaries of the large single-copy (LSC), small single-copy (SSC) and two inverted repeats (IR) regions was analyzed using IRscope (Amiryousefi et al., 2018). The nucleotide diversity (Pi) of shared genes and intergenic spacers was calculated using DnaSP (Librado and Rozas, 2009). The cp genomes of the 11 *Aristolochia* species together with those *Aristolochia* species available in NCBI, which were *A. bracteolata* (MT130705), *A. tagala* (NC041455), *A. debilis* (NC036153), *A. delavayi* (MW413320), *A. kaempferi* (NC041452), *A. mollissima* (NC041457), *A. kunmingensis* (NC041451), *A. moupinensis* (NC041454), *A. kwangsiensis* (NC052833) and *A. macrophylla* (NC041453), were used for phylogenetic analyses. The cp genomes of *Asarum pulchellum* (MZ440306) and *Piper kadsura* (NC027941) were included as the outgroup to root the tree. Considering the better-supported trees yielded by protein-coding data sets, a total of 72 protein-coding genes which were shared by these species were extracted to perform ML analysis using PhyloSuite software (Zhang et al., 2020; Guo et al., 2022). The maximum-likelihood (ML) analysis was performed based on the generated data using IQ-TREE with 1000 bootstrap replicates (Nguyen et al., 2015).

## 3 Results

### 3.1 Structure features of *Aristolochia* plastomes

**Figure 1:**
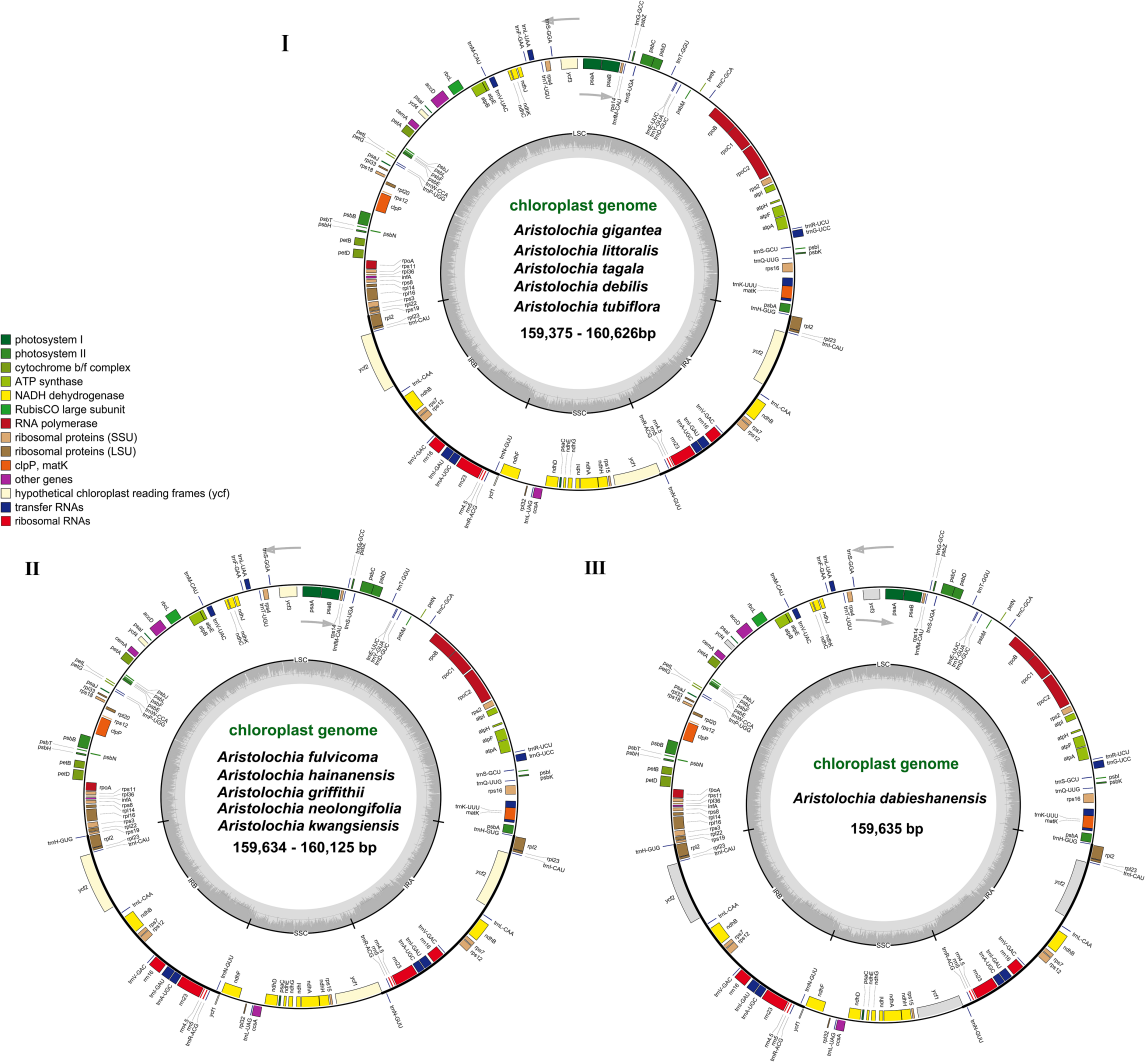
Gene maps of the complete cp genome of 11 species of Aristolochia. Three types of cp genome of **(I)** *A. gigantea, A. littoralis, A. tagala, A. debilis* and *A. tubiflora*. **(II)** *A. fulvicoma, A. hainanensis, A. griffithii, A. neolongifolia* and *A. kwangsiensis*. **(III)** *A. dabieshanensis*. Genes on the inside of the circle are transcribed clockwise, while those outside are transcribed counter clockwise. The darker gray in the inner circle corresponds to the GC content, whereas the lighter gray corresponds to AT content.

The complete cp genomes of 11 *Aristolochia* species were all typical quadripartite structures with the total length from 159,375 bp (*A. tagala*) to 160,626 bp (*A. tubiflora*) (**Figure 1**; **Table 1**). The consisted LSC region (88914-90251 bp) and SSC region (19311-19917 bp) were separated by two inverted repeat (IR) regions (50350-51396 bp) (**Table 1**). The total number of unique genes annotated is from 130 to 131, comprising 85 protein-coding genes (CDS), 37-38 tRNA and 8 rRNA genes (**Table 1**). GC contents of the plastomes of 11 *Aristolochia* species ranged slightly from 38.3% to 38.8%, and the GC contents of the four regions were not balanced. The IR regions had the highest GC content (43.4-43.6%), followed by the LSC regions (36.6-37.2%) and the SSC regions (32.8-33.8%) (**Supplementary Table 2**). The cumulative length of CDS ranged from 77,466 (*A. littoralis*) to 79,074 bp (*A. gigantea*) and the GC contents were 38.9% to 39.2% (**Table 1**; **Supplementary Table 2**). Moreover, the GC% content of the first position was higher compared to those of the second and third positions (**Supplementary Table 2**).

**Table 1:** Genome structure of complete chloroplast (cp) genomes of *Aristolochia* species.

Species	Genome Size(bp)	LSC(bp)	IR(bp)	SSC(bp)	CDS(bp)	Number of genes	rRNA	tRNA	Protein-coding genes	GC%	Genome type
*A. gigantea*	160594	90230	50854	19510	79074	130	8	37	85	38.4	I
*A. littoralis*	160610	90251	50886	19473	77466	130	8	37	85	38.4	I
*A. tagala*	159375	89441	50522	19412	78603	130	8	37	85	38.5	I
*A. debilis*	159812	89627	50350	19835	78708	130	8	37	85	38.3	I
*A. tubiflora*	160626	89945	50764	19917	78801	130	8	37	85	38.4	I
*A. fulvicoma*	159806	89200	51282	19324	78966	131	8	38	85	38.8	II
*A. hainanensis*	159672	89011	51340	19321	78954	131	8	38	85	38.8	II
*A. griffithii*	160125	89404	51386	19335	78921	131	8	38	85	38.7	II
*A. neolongifolia*	159634	88914	51396	19324	78948	131	8	38	85	38.8	II
*A. kwangsiensis*	159734	89069	51354	19311	78951	131	8	38	85	38.8	II
*A. dabieshanensis*	159635	88933	51362	19340	78942	131	8	38	85	38.8	III

### 3.2 Repeat structure and simple sequence repeats analyses

**Figure 2:**
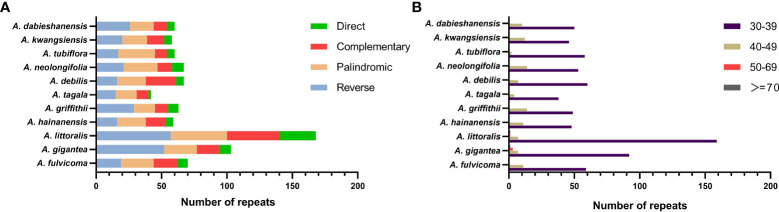
Long repeat sequences in the chloroplast genome of 11 species of *Aristolochia*. **(A)** Frequency of four types of repeats; **(B)** Length of repeat sequences.

A total of 817 repeats were identified in 11 *Aristolochia* species, including 288 reverse repeats, 260 palindromic repeats, 175 complement repeats, and 94 direct repeats (**Supplementary Table 3**). For each *Aristolochia* species, the number of repeat sequences varied greatly. *A. littoralis* had the largest number of repeats (168), while *A. tagala* had the smallest number of repeats (42). Four types of repeating motifs were detected in all 11 species (**Figure 2A**; **Supplementary Table 3**). The length of these repeats was mainly concentrated in 30-49 bp. Repeats with a length of ≥50bp only existed in *A. gigantea* and *A. littoralis* (**Figure 2B**; **Supplementary Table 4**).

**Figure 3:**
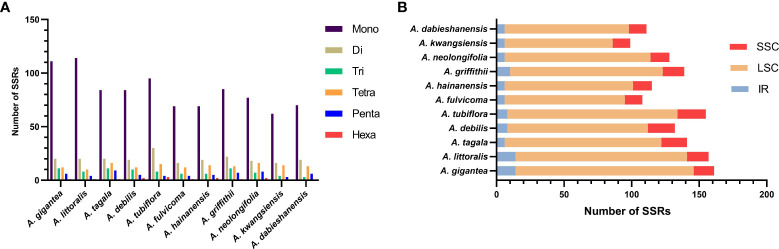
Amounts and distribution of SSRs in the chloroplast genome of 11 species of *Aristolochia*. **(A)** Amounts of the SSRs; **(B)** Distribution of SSRs.

Six kinds of SSRs were screened in the cp genomes of 11 *Aristolochia* species. The number of SSRs identified in 11 *Aristolochia* plastomes ranged from 99 in *A. kwangsiensis* to 161 in *A. gigantea* (**Supplementary Table 5**). In these SSRs, mono-nucleotide repeats were the largest in number, which accounted for the percent of 59.57%-72.61% in all types of SSRs (**Figure 3A**; **Supplementary Table 5**). The base composition of the repeating motifs had a certain base preference, mainly the repeating motifs rich in A-T (**Supplementary Table 5**). Eleven species all contained six kinds of repeat except for *A. kwangsiensis* and *A. dabieshanensis* which were without Hexa (**Figure 2A**; **Supplementary Table 5**). Regarding the SSRs distribution, these SSRs were mainly found in the LSC regions (**Figure 3B**; **Supplementary Table 6**).

### 3.3 Comparative genomic divergence and hotspots regions

**Figure 4:**
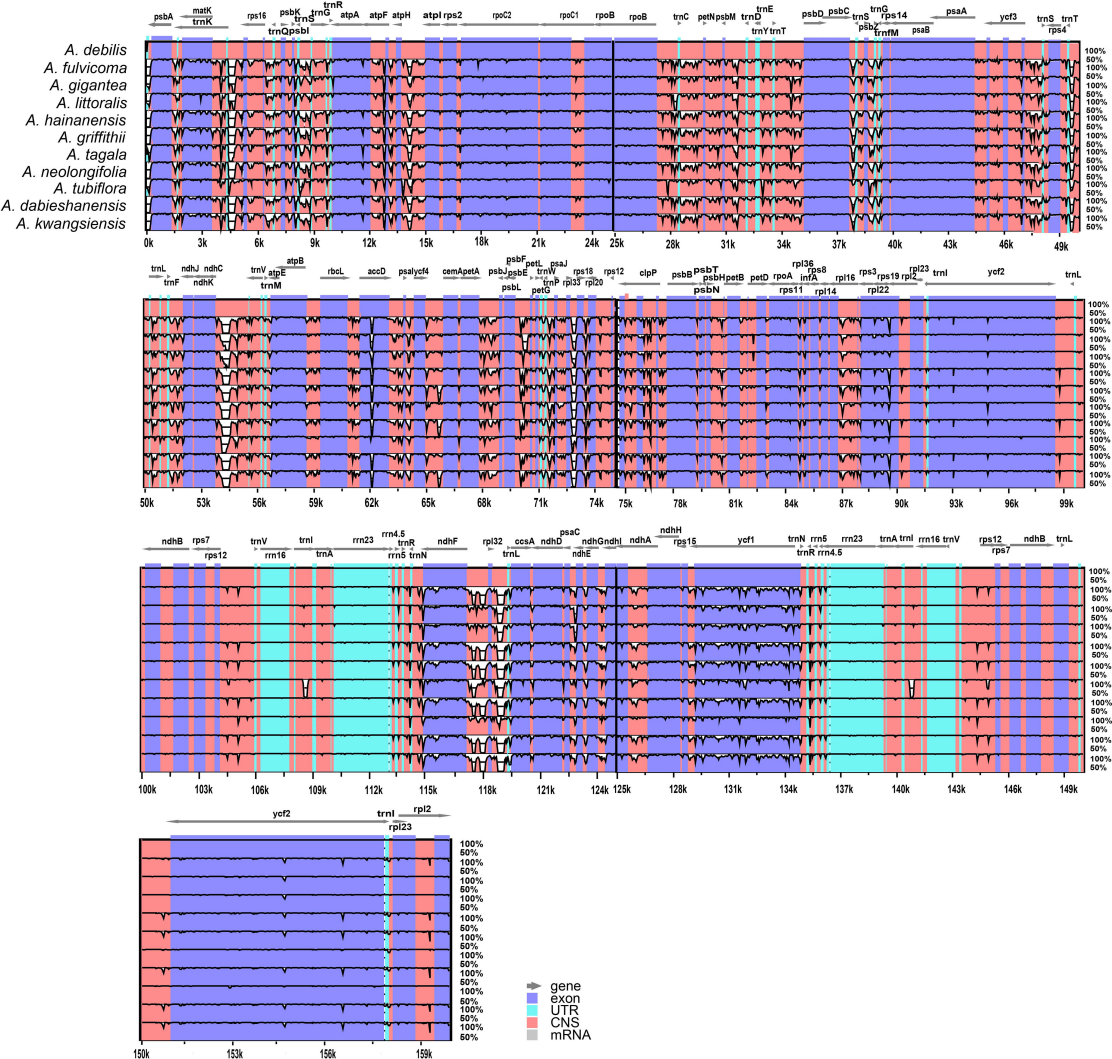
Sequence identity plot comparing the four chloroplast genomes of species of *Aristolochia* with *Aristolochia debilis* as a reference using mVISTA. Gray arrows and thick black lines above the alignment indicate genes with their orientation. Purple bars represent exons, blue bars represent UTRs, and red bars represent noncoding sequences (CNS). Y-scale represents the percent identity ranging from 50% to 100%.

**Figure 5:**
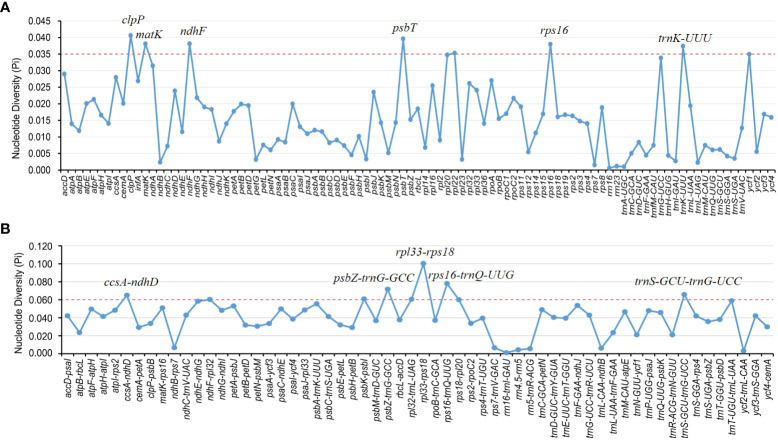
Nucleotide diversity (Pi) of shared various regions in 11 *Aristolochia* species chloroplast genomes. **(A)** Pi values in the genes regions. **(B)** Pi values in the intergenic spacers regions.

Divergence hotspots are important for discovering DNA markers and barcodes in species identification (Kong et al., 2021). In this study, the cp genomes of 11 species of *Aristolochia* were compared using mVISTA with the *A. debilis* genome as the reference genome. Overall, the comparative genomic analysis revealed that the 11 *Aristolochia* cp genomes were relatively conserved. Most variations are discovered in the conserved noncoding sequences, and only a few in coding genes, such as *accD*, *ndhF* and *ycf1* (**Figure 4**). The results indicated that the coding-gene sequences were more conserved than the noncoding sequences. Moreover, the nucleic acid variation analyses showed the intergenic spacers had more polymorphisms (average Pi=0. 04049) than the gene regions (average Pi=0.01546) (**Figure 5**). The highly variable regions comprised the genes regions: *clpP*, *matK*, *ndhF*, *psbT*, *rps16*, *trnK*-UUU (Pi>0.035). Among the six highly variable regions, five regions *clpP*, *matK*, *psbT*, *rps16*, and *trnK-*UUU were located in the LSC, and *ndhF* was located in the SSC. The intergenic spacer regions with high variations were screened as follows: *ccsA-ndhD*, *psbZ-trnG-*GCC, *rpl33-rps18*, *rps16-trnQ-*UUG, *trnS-*GCU*-trnG-*UCC (Pi>0.060). Among the five highly variable regions, four regions, *rpl33-rps18*, *rps16-trnQ-*UUG, *psbZ-trnG-*GCC, and t*rnS-*GCU*-trnG-*UCC were located in the LSC, and *ccsA-ndhD* were located in the SSC. It was confirmed that the variations in the LSC and SSC regions were remarkably higher than those in the IR regions of cp.

### 3.4 Phylogenetic analyses

**Figure 6:**
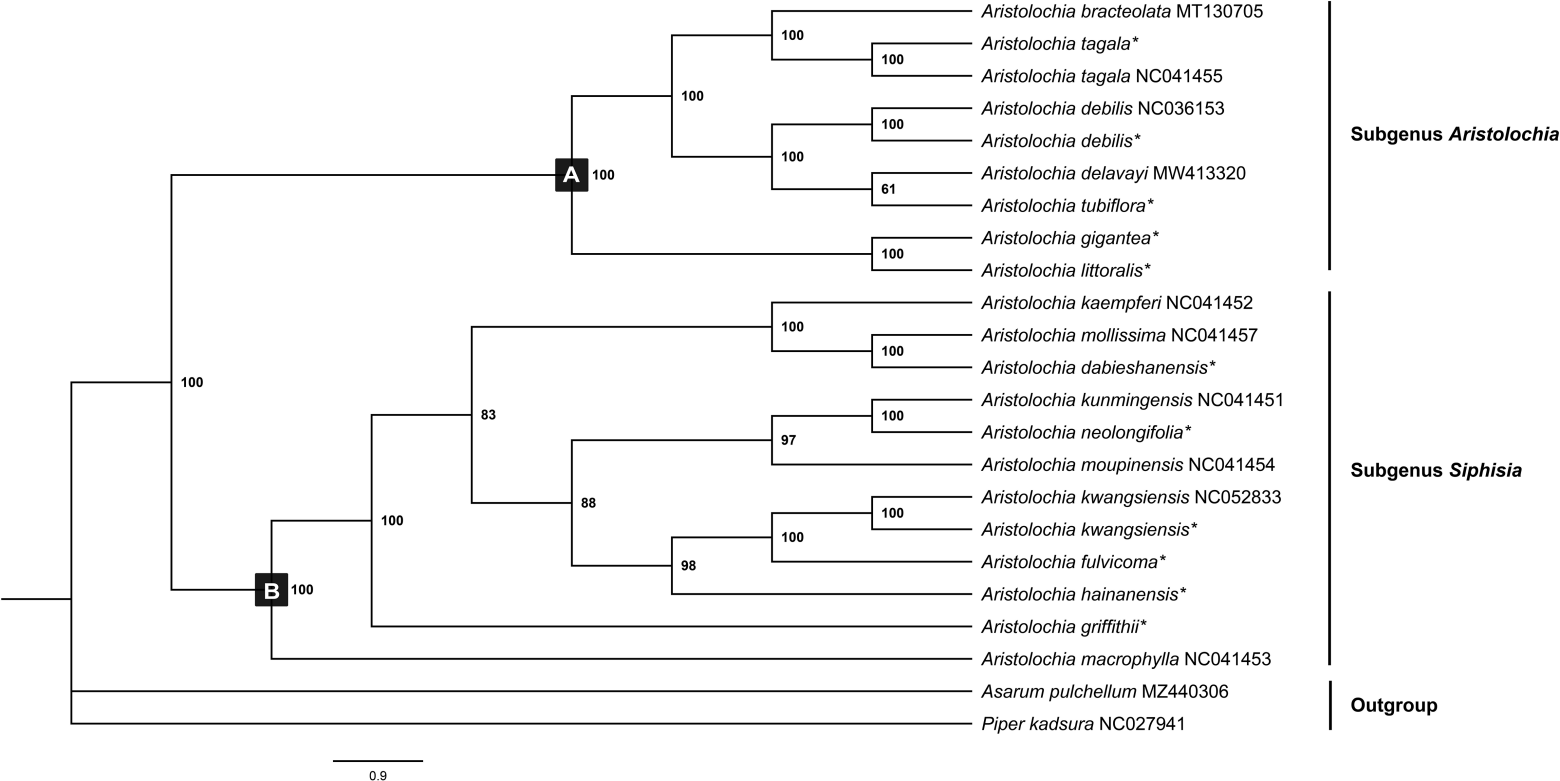
Phylogenetic tree inferred from the CDS of the 72 protein-coding genes of *Aristolochia* species using Maximum Likelihood (ML) method. The numbers near by nodes are values for bootstrap support. Species with newly sequenced chloroplast genomes are marked with asterisks.

Chloroplast genomes play an important role in phylogenetic studies, and it is necessary to solve complex evolutionary relationships (Zhang et al., 2011). In our study, to obtain a more accurate analysis of the *Aristolochia* phylogeny, available *Aristolochia* genomes downloaded from NCBI were also included to construct the phylogenetic tree. A total of eighteen *Aristolochia* species were contained, and *Asarum pulchellum* and *Piper kadsura* served as the outgroup (**Figure 6**). Phylogenetic analyses using the ML method and sequences of 72 CDS strongly supported the identification of two clades among *Aristolochia* species, and they corresponded to subgenus *Aristolochia* (Clade A) and subgenus *Siphisia* (Clade B), as classified in *Flora of China* (Huang et al., 2003). Within the subgenus *Aristolochia*, *A. gigantea* and *A. littoralis* formed a monophyletic cluster, which was a sister to the other five *Aristolochia* species (*A. bracteolate*, *A. tagala*, *A. delavayi*, *A. tubiflora* and *A. debilis*). In subgenus *Siphisia*, *A. macrophylla* diverged first. Then *A. griffithii* showed a sister relationship with remaining *Siphisia* species. The monophyletic cluster comprising *A. fulvicoma*, *A. kwangsiensis* and *A. hainanensis* was a sister to the cluster composed of *A. kunmingensis*, *A. neolongifolia* and *A.moupinensis*, and both were sister to the other three species (*A. kaempferi*, *A. mollissima* and *A. dabieshanensis*).

### 3.5 IR expansion and contraction investigation

**Figure 7:**
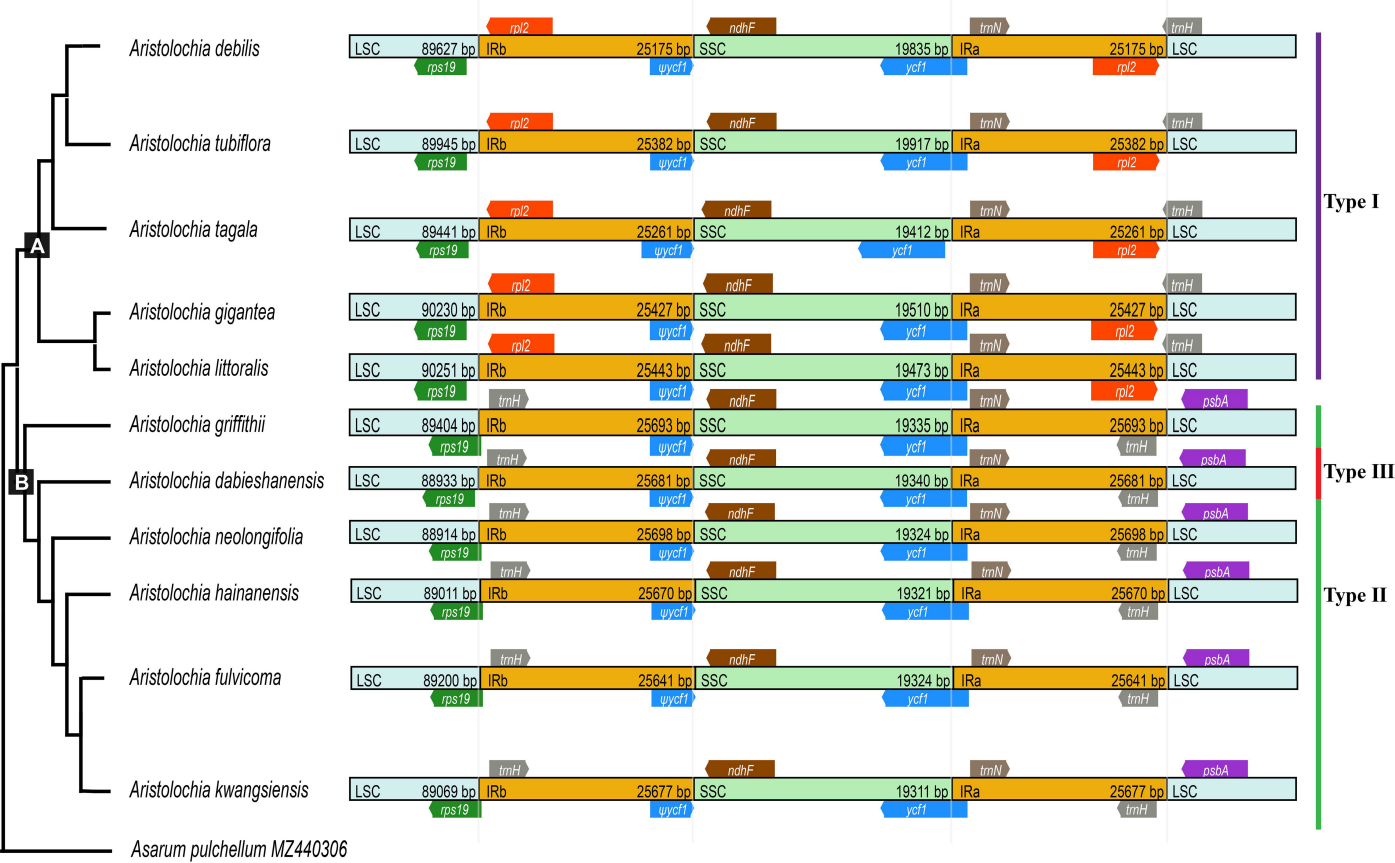
Comparison of boundaries of the LSC, SSC, and IR regions among 11 *Aristolochia* plastomes.

The boundaries of IR region are hot spots for gene duplications or deletions (Yue et al., 2008). In this study, the expansion and contraction of the IR region in 11 *Aristolochia* cp genomes were analyzed. Results showed that all *Aristolochia* plastomes have the SSC/IRb boundary within the pseudogene (ψ) *ycf1* gene and the SSC/IRa border within the *ycf1* gene except the *A. tagala* which between the *ycf1* and gene *trnN* (**Figure 7**). However, there were some differences in the IR/LSC border area, and three types of plastomes were characterized by IR/LSC boundary variation (**Figures 1**, **7**). *A. debilis*, *A. tubiflora*, *A. tagala*, *A. gigantea* and *A. littorali* were grouped together in Clade A and classified as Type I, because the LSC-IRb border of cp genomes was located within the genic spacer of *rps19-rpl2* as well as the LSC-IRa border was located within the gene *trnH*. Type II and III corresponded to Clade B which contained one more repeat of *trnH*-GUG in the IRb region and the LSC-IRb border was located within the gene *rps19* (*A. fulvicoma, A. hainanensis, A. griffithii, A. neolongifolia* and *A. kwangsiensis*) or the genic spacer of *rps19-trnH* (*A. dabieshanensis*). Besides, the IRa regions of Type II and III had slightly expanded, resulting in *trnH* being located in the IR region, and the IRa/LSC border was located in the genic spacer of *trnH*-*psbA*. Compared with the type I, the IR region of the remaining two types of plastomes expanded approximately 0.4-1.0 k (**Table 1**; **Figure 7**).

## 4 Discussion

In this study, we reported eleven sequenced complete cp genomes of *Aristolochia* (**Figure 1**; **Table 1**). Our comparative analyses indicated that the overall eleven cp genomes showed a highly conserved feature in terms of structures. The GC content of eleven *Aristolochia* plants ranged from 38.3% to 38.8%, which was the same as that previously reported *Aristolochia* plastome (Zhou et al., 2017; Li et al., 2019). Besides, the IR regions had the highest GC content among the four regions of these *Aristolochia* species, which was consistent with most other angiosperms (Wu et al., 2020). SSRs, also known as cp microsatellites, were short tandem repeat sequences consisting of 1-6 bp nucleotides as repeating units. In all types of SSRs in this study, A or T repeats accounted for the majority, and mono-nucleotides were the predominant type. The richness of A/T in cp genomes can be explained by the easier strand separation for increasing the slipped-strand mispairing as compared to GC/CG and other tracts (George et al., 2015). Widely distributed SSRs in cp genomes provide the available molecular markers for the species of interest or closely related species (Varshney et al., 2005; Vu et al., 2020). In orchids, SSR markers were developed for recognizing valuable plants, investigating intraspecific genetic variation and reconstructing phylogeographic patterns (Tsa et al., 2014). The SSRs detected in the *Aristolochia* species were of great significance for the phylogenetic research and classification of *Aristolochia* plants. Additionally, four types of long repeat sequences were all identified in 11 *Aristolochia* species including direct, reverse, complement and palindromic. Most of the repeats were reversed and palindromic. These long repeat sequences were not only abundant in mutations but also very important in phylogenetic analyses (Wu et al., 2021). All the identified repeats in this study may be useful for the population genetics studies of these 11 species in the future.

Although the cp genomes among 11 *Aristolochia* species have a highly conserved feature, there were some small changes presented among these species on the boundary between the IR and LSC regions. Plastid genomes have been divided into three types according to the boundary of LSC/IR, which has a certain relationship corresponding to the clades of two subgenera *Aristolochia* and *Siphisia*. By comparing the length variation of IR, LSC and SSC regions among these cp genomes, we also found that the IR region of the plastomes of subgenus *Siphisia* expanded approximately 0.4-1.0 k to the LCS region compared to the subgenus *Aristolochia* (**Figure 7**). The expansions and contractions of the boundaries of the IR regions are considered to be the main reason for the size change of cp (Zhang et al., 2016). Besides, the deletion of one copy of *trnH*-GUG gene was observed in subgenus *Aristolochia* species, which resulted in the total of 37 tRNA genes in the species of subgenus *Aristolochia* and 38 in *Siphisia* (**Table 1**). A previous study also reported the loss of the *trnH*-GUG genes was one of the major differences between the plastomes of the two subgenera *Siphisia* and *Aristolochia* (Li et al., 2019). These sequence variations might be the result of boundary contraction and expansion between the LSC/IR regions in plants (Wang et al., 2022). Plastid genomes have the characteristics of high conservation and slow evolutionary rate, thus the special characteristics presented in their structure are often phylogenetically informative (Pascual-Diaz et al., 2021). In general, broad sampling and more evidence from the genomes will be necessary for the further understanding of the interspecies relationships of *Aristolochia*.

Species of *Aristolochia* are controversially officinal and strictly forbidden in the present. The identification of *Aristolochia* species is important to supervise the abuse and protect customer safety. Morphological evidence is a conventional method for plant classification and identification. However, morphological traits are easily affected by the natural environment and artificial treatment, which hardly meet the requirements of detection in practical application. DNA studies can achieve the accurate authentication of similar species within a genus based on reliable molecular evidence. Numerous DNA regions, such as the nuclear genes ITS2, and cp genes *matK*, *rbcL*, *trnH-psbA* and *trnL-trnF*, have been applied to the identification of *Aristolochia* species (Li et al., 2014; Dechbumroong et al., 2018). However, multiple primers were required to achieve the authentication of different *Aristolochia* species, and the existence of long sequence deletions or poly-A/T sequences also resulted in the difficulty of sequencing analysis (Wu et al., 2015). In this study, the results of mVISTA analysis suggested that the hypervariable intergenic regions were mostly distributed in the non-coding regions, and rarely in coding genes. Moreover, our comparative results have shown that the used cp markers appeared to be relatively low in nucleotide diversity, which may be insufficient to distinguish the species within genus *Aristolochia.* Thus, to achieve better species resolution, future molecular markers can focus on the more variable regions of the cp genomes, such as *clpP*, *psbT*, *rps16, ycf1* and *rpl33-rps18* (Chang et al., 2021).

With increasing taxon samples of *Aristolochia* species, our phylogenetic analyses of cp genome sequences have substantially improved the phylogenetic resolution and provided robust inference of the intraspecific relationships. In the current study, phylogenetic trees of the genus *Aristolochia* were constructed based on CDS sequences from a total of 18 *Aristolochia* species, including eleven species we sequenced and other seven downloaded from NCBI. Regarding the division of genus *Aristolochia*, our phylogenetic analyses have confirmed the division of two clades representing the species of subgenus *Aristolochia* and *Siphisia*, respectively. This cp phylogeny concurs well with previously published phylogenetic trees based on several nuclear/plastid regions (Zhu et al., 2019a). Compared with the phylogenetic results, it is further confirmed that the species clustered in subgenus *Siphisia* also could be corresponded with the *Isotrema* species, which is consistent with the classification based on the morphological characteristics, number of chromosomes and molecular data (Huang et al., 2003; Ohi-Toma et al., 2006; Zhu et al., 2019b). Our result provided stronger support that the subgenus *Siphisia* was clustered as an independent clade, and may contribute to the reinstatement of *Isotrema* as a new generic delimitation of *Aristolochia* subgenus *Siphisia*. In general, the phylogenetic tree conducted in this study demonstrated that the cp genomes can be used as essential evidence to resolve the intergeneric and interspecies relationships within genus *Aristolochia*.

## 5 Conclusion

In this study, the complete cp genomes of eleven species of genus *Aristolochia* were sequenced and compared. All of these cp genomes were obvious quadripartite structures and comparatively conserved on the length, GC content and gene content. The high variations were mostly found in LCS and SSC regions, and variable regions could serve as potential markers for species identification. Phylogenetic results indicated that the genus *Aristolochia* was composed of two main clades, corresponding to the division of subgenus *Siphisia* and subgenus *Aristolochia*. Moreover, combined with the analyses of IR/LSC boundaries, a whole duplication of *trnH*-GUG gene was observed in subgenus *Siphisia*, and it may be associated with the expansion of its IR region. In conclusion, this study provides an important foundation for species identification and valuable insight into the phylogenetic relationships of the *Aristolochia*.

## Data availability statement

The data presented in the study are deposited in the NCBI repository (https://www.ncbi.nlm.nih.gov/), and the accession numbers were OP895634, OP925753, OP950686-OP950694.

## Author contributions

JH, XB, GW, YR and YS conceived and designed the study. XB, GW collected and analyzed the data. XB, YR and YS wrote the manuscript. All authors have directly contributed to this manuscript. All authors contributed to the article and approved the submitted version.
