# Supplementary material for: Insights into taxonomy and phylogenetic relationships of eleven Aristolochia species based on chloroplast genome
Source: Front Plant Sci. 2023 Feb 13;14:1119041. doi: 10.3389/fpls.2023.1119041 (PMC9969298; doi:10.3389/fpls.2023.1119041)
Supplement: Supplementary file 1 [file Table_1.docx]

Supplementary Material

Supplementary Table S1 Voucher information and GenBank accession numbers for the *Aristolochia* species we sequenced.

| **Species** | **Voucher No.** | **Accession No.** | | **Location** | **Sampling Part** |
| --- | --- | --- | --- | --- | --- |
| *A. gigantea* | JH | | OP925753 | Mengla County, Xishuangbanna autonomous prefecture, Yunnan Province | leaf |
| *A. littoralis* | ML | | OP950686 | Mengla County, Xishuangbanna autonomous prefecture, Yunnan Province | leaf |
| *A. tagala* | EY | | OP950689 | Tongtieling Mountain, Wanning City, Hainan Province | leaf |
| *A. debilis* | MDL | | OP950690 | Dangwu Town, Guiyang City, Guizhou Province | leaf |
| *A. tubiflora* | GH | | OP950692 | Bajiao villages, Jianshi County,Enshi City, Hubei Province | leaf |
| *A. fulvicoma* | HH | | OP895634 | Tongtieling Mountain, Wanning City, Hainan Province | leaf |
| *A. hainanensis* | HN | | OP950687 | Mengla County, Xishuangbanna autonomous prefecture, Yunnan Province | leaf |
| *A. griffithii* | XZ | | OP950688 | Chentang Town,Shigatse City, Tibet | leaf |
| *A. neolongifolia* | XY | | OP950691 | —— | leaf |
| *A. kwangsiensis* | GX | | OP950693 | Bajiao villages, Jianshi County,Enshi City, Hubei Province | leaf |
| *A. dabieshanensis* | DBS | | OP950694 | Dabie Mountain, Luotian County,Huanggang City, Hubei Province | leaf |

Supplementary Table S2 GC content for chloroplast genomes of the eleven *Aristolochia* species

|  |  | **GC(%)** | | | | | | | | | | |
| --- | --- | --- | --- | --- | --- | --- | --- | --- | --- | --- | --- | --- |
|  |  | *A. gigantea* | *A. littoralis* | *A. tagala* | *A. debilis* | *A. tubiflora* | *A. fulvicoma* | *A. hainanensis* | *A. griffithii* | *A. neolongifolia* | *A. kwangsiensis* | *A. dabieshanensis* |
| Total |  | 38.4 | 38.4 | 38.5 | 38.3 | 38.4 | 38.8 | 38.8 | 38.7 | 38.8 | 38.8 | 38.8 |
| IRa |  | 43.4 | 43.4 | 43.4 | 43.4 | 43.5 | 43.6 | 43.5 | 43.5 | 43.5 | 43.5 | 43.5 |
| IRb |  | 43.4 | 43.4 | 43.4 | 43.4 | 43.5 | 43.6 | 43.5 | 43.5 | 43.5 | 43.5 | 43.5 |
| LSC |  | 36.8 | 36.8 | 36.8 | 36.6 | 36.7 | 37.1 | 37.2 | 37.0 | 37.1 | 37.2 | 37.2 |
| SSC |  | 33.2 | 33.2 | 33.1 | 32.8 | 32.9 | 33.8 | 33.8 | 33.7 | 33.8 | 33.8 | 33.8 |
| CDS |  | 38.9 | 39.0 | 38.9 | 38.9 | 38.9 | 39.2 | 39.2 | 39.2 | 39.2 | 39.2 | 39.2 |
|  | 1st position | 46.0 | 46.1 | 45.9 | 46.0 | 45.9 | 46.3 | 46.3 | 46.3 | 46.3 | 46.3 | 46.3 |
|  | 2nd position | 38.7 | 38.8 | 38.7 | 38.7 | 38.7 | 38.9 | 38.9 | 38.9 | 38.9 | 38.9 | 38.9 |
|  | 3rd position | 32.0 | 32.0 | 32.0 | 32.0 | 32.2 | 32.4 | 32.4 | 32.4 | 32.4 | 32.4 | 32.4 |

Supplementary Table S3 Types and amounts of repeats in the *Aristolochia* plastomes.

| **Species** | **Reverse** | **Palindromic** | **Complementary** | **Forward** | **Total** |
| --- | --- | --- | --- | --- | --- |
| *A. fulvicoma* | 19 | 25 | 18 | 8 | 70 |
| *A. gigantea* | 52 | 25 | 17 | 9 | 103 |
| *A. littoralis* | 57 | 43 | 40 | 28 | 168 |
| *A. hainanensis* | 16 | 22 | 15 | 6 | 59 |
| *A. griffithii* | 29 | 16 | 10 | 8 | 63 |
| *A. tagala* | 15 | 16 | 9 | 2 | 42 |
| *A. debilis* | 16 | 22 | 23 | 6 | 67 |
| *A. neolongifolia* | 21 | 26 | 11 | 9 | 67 |
| *A. tubiflora* | 17 | 28 | 9 | 6 | 60 |
| *A. kwangsiensis* | 20 | 19 | 13 | 6 | 58 |
| *A. dabieshanensis* | 26 | 18 | 10 | 6 | 60 |
| Total | 288 | 260 | 175 | 94 | 817 |

Supplementary Table S4 Different length of repeats in the *Aristolochia* plastomes.

| **Species** | **30-39** | **40-49** | **50-59** | **60-69** | **>=70** |
| --- | --- | --- | --- | --- | --- |
| *A. gigantea* | 92 | 7 | 3 | 0 | 1 |
| *A. littoralis* | 159 | 7 | 1 | 0 | 1 |
| *A. tagala* | 38 | 4 |  |  |  |
| *A. debilis* | 60 | 7 |  |  |  |
| *A. tubiflora* | 58 | 2 |  |  |  |
| *A. fulvicoma* | 59 | 11 |  |  |  |
| *A. hainanensis* | 48 | 11 |  |  |  |
| *A. griffithii* | 49 | 14 |  |  |  |
| *A. neolongifolia* | 53 | 14 |  |  |  |
| *A. kwangsiensis* | 46 | 12 |  |  |  |
| *A. dabieshanensis* | 50 | 10 |  |  |  |

| Supplementary Table S5 Types and amounts of SSRs in the *Aristolochia* plastomes. | | | | | | | | | | | | |
| --- | --- | --- | --- | --- | --- | --- | --- | --- | --- | --- | --- | --- |
| SSR type | Repeat units | species | | | | | | | | | | |
|  |  | *A. fulvicoma* | *A. gigantea* | *A. littoralis* | *A. hainanensis* | *A. griffithii* | *A. tagala* | *A. debilis* | *A. neolongifolia* | *A. tubiflora* | *A. kwangsiensis* | *A. dabieshanensis* |
| Mono | A/T | 65 | 110 | 113 | 65 | 82 | 79 | 80 | 72 | 89 | 59 | 65 |
|  | C/G | 4 | 1 | 1 | 4 | 3 | 5 | 4 | 5 | 6 | 3 | 5 |
| Di | AT/AT | 16 | 19 | 19 | 19 | 20 | 20 | 19 | 18 | 29 | 16 | 19 |
|  | AG/CT | - | 1 | 1 | - | 2 | - | - | - | - | - | - |
|  | AC/GT | - | - | - | - | - | - | - | - | 1 | - | - |
| Tri | AAT/ATT | 6 | 9 | 6 | 6 | 11 | 9 | 7 | 7 | 6 | 4 | 3 |
|  | AAC/GTT | - | 1 | 1 | - | - | 1 | 1 | - | 1 | - | - |
|  | AAG/CTT | - | 1 | 1 | - | - | 1 | 1 | - | - | - | - |
|  | AGG/CCT | - | - | - | - | - | - | - | - | 1 | - | - |
|  | ATC/ATG | - | - | - | - | - | - | 1 | - | - | - | - |
| Tetra | AAAC/GTTT | 2 | 2 | 1 | 2 | 2 | 3 | 2 | 2 | 2 | 2 | 2 |
|  | AAAG/CTTT | 1 | 1 | 1 | 1 | 1 | 1 | - | 1 | 1 | 1 | 1 |
|  | AAAT/ATTT | 5 | 7 | 6 | 5 | 4 | 7 | 4 | 7 | 7 | 6 | 4 |
|  | AATC/ATTG | 1 | - | - | 1 | 1 | 1 | 1 | 1 | 1 | 1 | 1 |
|  | AATG/ATTC | 2 | 1 | 1 | 3 | 3 | 2 | 2 | 3 | 1 | 2 | 3 |
|  | AGAT/ATCT | 1 | - | - | 2 | 2 | - | 2 | 2 | 1 | 2 | 2 |
|  | AACT/AGTT | - | 1 | 1 | - | - | 1 | 1 | - | 1 | - | - |
|  | AATT/AATT | - | - | - | - | - | - | - | - | 1 | - | - |
|  | ACAT/ATGT | - | - | - | - | - | 1 | - | - | - | - | - |
| Penta | AAAAT/ATTTT | 1 | 2 | 2 | 1 | 1 | 1 | 1 | 3 | - | 1 | 2 |
|  | AAATG/ATTTC | 1 | - | - | 1 | 1 | - | - | 1 | - | 1 | 1 |
|  | AATAT/ATATT | 2 | 4 | 2 | 1 | 3 | 3 | 2 | 2 | 3 | - | 2 |
|  | AATAC/ATTGT | - | - | - | 1 | 2 | - | - | - | 1 | 1 | - |
|  | AAAAG/CTTTT | - | - | - | 1 | - | - | - | - | - | - | - |
|  | AAATC/ATTTG | - | - | - | - | - | - | 1 | - | - | - | - |
|  | AGCAT/ATGCT | - | - | - | - | - | 2 | - | - | - | - | - |
|  | AAATT/AATTT | - | - | - | - | - | 3 | 1 | - | - | - | - |
|  | ATATC/ATATG | - | - | - | - | - | - | - | 2 | - | - | 1 |
| Hexa | AAAATT/AATTTT | 1 | - | - | 1 | - | 1 | - | - | - | - | - |
|  | AAAAAT/ATTTTT | - | - | - | - | - | - | 1 | - | - | - | - |
|  | ACTGAT/AGTATC | - | - | - | - | - | - | 1 | - | - | - | - |
|  | AAAGAT/ATCTTT | - | - | - | - | - | - | - | 1 | - | - | - |
|  | AAAATG/ATTTTC | - | - | - | - | - | - | - | 1 | - | - | - |
|  | AAATAT/ATATTT | - | - | - | 1 | - | - | - | - | - | - | - |
|  | AATAGT/ACTATT | - | - | - | - | 1 | - | - | - | - | - | - |
|  | AATTAT/AATTAT | - | 1 | 1 | - | - | - | - | - | - | - | - |
|  | AAATAG/ATTTCT | - | - | - | - | - | - | - | - | 1 | - | - |
|  | ACTCTC/AGAGTG | - | - | - | - | - | - | - | - | 1 | - | - |
|  | AAAAAG/CTTTTT | - | - | - | - | - | - | - | - | 1 | - | - |
| Total |  | 108 | 161 | 157 | 115 | 139 | 141 | 132 | 128 | 155 | 99 | 111 |

Supplementary Table S6 Distribution of SSRs in the *Aristolochia* plastomes.

| **Species** | **IR** | **LSC** | **SSC** | **Total** |
| --- | --- | --- | --- | --- |
| *A. gigantea* | 14 | 132 | 15 | 161 |
| *A. littoralis* | 14 | 127 | 16 | 157 |
| *A. tagala* | 6 | 116 | 19 | 141 |
| *A. debilis* | 8 | 104 | 20 | 132 |
| *A. tubiflora* | 8 | 126 | 21 | 155 |
| *A. fulvicoma* | 6 | 89 | 13 | 108 |
| *A. hainanensis* | 6 | 95 | 14 | 115 |
| *A. griffithii* | 10 | 113 | 16 | 139 |
| *A. neolongifolia* | 6 | 108 | 14 | 128 |
| *A. kwangsiensis* | 6 | 80 | 13 | 99 |
| *A. dabieshanensis* | 6 | 92 | 13 | 111 |
